# Synthesis and characterization of flame retardant unsaturated polyester-allyloxysilane resin for wood coatings

**DOI:** 10.1038/s41598-024-62765-x

**Published:** 2024-06-11

**Authors:** Iman Mohammadi Dehcheshmeh, Ahmad Poursattar Marjani, Fatemeh Sadegh, Mohammad Ebrahim Soltani, Mohammad Safaeirad, Marco Frediani

**Affiliations:** 1 Department of Organic Chemistry, Faculty of Chemistry, Urmia University, Urmia, Iran; 2 Department of Chemistry “Ugo Schiff”, University of Florence, Via della Lastruccia, 3-13, 50019 Sesto Fiorentino, Florence, Italy; 3 Department of Chemistry, Faculty of Sciences, University of Sistan and Baluchestan, Zahedan, Iran; 4 Department of Chemical Engineering, Isfahan University of Technology, Isfahan, 8415683111, Iran; 5 Tuka Paint Foolad Sepahan Industrial and Manufacturing Company Unit Resin Research and Development Center, Shahreza, Isfahan, Iran

**Keywords:** Unsaturated polyester, Flame retardant, Wood coatings, Tetraallyloxysilane, Resin, Polymer chemistry, Polymer synthesis

## Abstract

Fireproof coatings are the simplest, most efficient, and oldest method for protecting a wide range of flammable products, such as wood. Furthermore, surface ignition is the initial phase, so surface protection is essential to reduce fire propagation. Furthermore, delaying the spread of flames can help to save someone when a fire starts. This project synthesized flame-resistant resin starting from tetraallyloxysilane monomer as a halogen-free monomer, an intrinsic flame retardant co-curing agent. The following step synthesized polyester resin using terephthalic acid as a heat-resistant resin. Unsaturated polyester was used by bulk radical polymerization. FT-IR and ^1^H-NMR analysis showed the successful synthesis of the desired monomer and polymeric compound. The thermal degradation and flame retardancy of pure unsaturated polyester resin (UPE) and allyloxysilane-unsaturated polyester (AUPE) were investigated by thermogravimetric analysis (TGA/DTG/DSC). The burning test and the thermal stability of the coating layers were evaluated using standard UL 94. Physical properties of resins were evaluated using Heat Deflection Temp tests (HDT) ISO 75-A, ASTM 648, Hardness ASTM D2583, Volumetric shrinkage ASTM 3521, and Water absorption ASTM D570. The results of the tests show the successful synthesis and their flame retardant properties.

## Introduction

Wood is a flexible and lightweight material mainly composed of hemicellulose, cellulose, and lignin. It is widely used to realize interior decoration and building frames due to its renewable nature, easy and cheap access, natural durability, and complicated and robust structure ^1,2^. All over the world, the damages and injuries caused by frequent fires inside or outside the building have caused many casualties. In the survey of 3.3 billion people in 2020 in 48 countries of the world, 69.5 thousand people were injured, and 20.6 thousand of them lost their lives during the fire^3^. With the ever-increasing use of wood and the widespread density of houses in densely populated countries, fire hazards of using wood as inherent flammability significantly exacerbate^4–6^. Therefore, the use of flame retardant coatings can be beneficial. These coatings can be realized using a physical mixture (additive) or a reactive product linked to the wood substrate.

In most cases, reactive products are preferred over additive mixing in the coating as they will have a longer shelf life^7–10^. Several flame retardants, such as halogen-based retardants^11,12^, phosphorus^13–18^, nitrogen^19–21^, boron^22–24^, biochar^25,26^, metal oxides^27,28^, are currently employed. Currently, halogens are one of the most widely used. Their flame retardancy is excellent, but with the production of toxic gases, the bioaccumulation of halogens has affected human health, and limiting and banning their use has been announced worldwide^29^. Ideal fireproof coatings should have characteristics such as reducing flame spread, low emission of smoke and toxic gas, ease of use, adhesion to the surface, and low cost^30^. As a thermoset material, durable, strong, easy to dry, and excellent adhesion, polyesters are one of the most widely used resins in the coating industry. They are used for various applications^31–34^. The cause of flame retardancy in polyesters is its chemical composition^35–38^. Advances in fire-resistant coatings have been made since 2020 for polyester resin, offering better performance and efficiency in protecting materials from fire hazards. These new coatings use innovative formulations and technologies to enhance fire resistance and safety measures. Nanotechnology has been widely used for polyester resin in developing fire-resistant coatings^39^. Nanoparticles such as carbon nanotubes^40,41^, graphene oxide^42^, and nanoclay^43^ are included in coatings to increase fire resistance properties. New coatings with multipurpose properties, called multipurpose fireproof coatings, are being developed to combine fireproofing with other capabilities such as UV resistance, anticorrosion, and self-healing capabilities^44^, but these coatings are not cost-effective and transparent. Not being, they have not yet been able to be commercialized as final wood coatings. When comparing these new fire-resistant coatings for polyester resin, factors such as fire resistance performance, durability, stability, smart features, ease of application, and cost-effectiveness should be considered. Choosing the most appropriate option to increase fire safety by conducting a detailed analysis of these coatings based on specific performance requirements and criteria can help in various applications of polyester resin materials^45^.

In this project, an attempt has been made to synthesize unsaturated polyester resin by selecting terephthalic acid as a heat-resistant compound in the polymer body. Then, all the properties mentioned above were improved by synthesizing triethylsilane and adding it to the unsaturated polyester, obtaining the desired resin.

## Experimental

### Materials

Tetraethyl orthosilicate (TEOS) by Coupleshimi Sepahan (Iran), NaOH by Nirouchlor (Iran), Dibutyl tin oxide (DBTO), Allyl chloride, Terephthalic acid (PTA), Ethanol, benzoyl peroxide, Maleic Anhydride (MA), and Tetrahydrofuran (THF) by Aldrich chemicals (USA), Ethylene glycol (EG) by shazand petrochemical company (Iran), Styrene by pars petrochemical company (Iran), Cobalt octoate 10% as an oxidation catalyst by shimie afsoon company (Iran).

### Instruments

Fourier-transform infrared (FTIR) spectra were recorded between 400 and 4000 cm^−1^ Nicolet model Impact 400D (Madison, USA); ^1^H-NMR spectra using a CDCl_3_ solution with a spectrometer Bruker Avance III 400, at room temperature using Tetramethlysilane (TMS) as internal standard. In these spectra, the number of branches and hydrogens or each peak are indicated using the Latin alphabet in the figure and text. Single peaks are shown by “s”, double peaks by “d”, triple peaks by “t”, quartet peaks by “q”, and multiple peaks by “m”.

Thermal analysis (TGA) of polyester resins made by differential scanning calorimeter (DSC, Mettler TA 4000, New Zealand) from − 100 to 100 °C at a temperature rate of 20 °C/min under nitrogen gas, in the form of went back and forth, and thermal analysis of the resins made before and after curing was done under nitrogen gas at a rate of 20 °C/min by Mettler Toledo TGA model SOTA 851e (Switzerland).

Gel permeation chromatography was employed to detect the molecular weight of the polymers using an analytical scientific instrument, GPC Model 500 (USA), equipped with a refractive index detector calibrated by polystyrene having a standard molecular mass using tetrahydrofuran solution.

Sample topography was carried out using a microscope Model 10 Microns (0.01) Sheen.

### Flammability method and standards

Underwriters Laboratories (UL) of the United States have published the UL 94 standard to determine the flammability of plastic materials. It shows the ability of plastic parts to spread or extinguish the flame. A radiant heat source or a small open flame can also assess its burning behavior in controlled experimental conditions. This test can be an initial indication to check the flammability of plastics for various applications, such as polymers for surface coatings and plastic parts used in electric and electronic devices. This test evaluates the burning in the vertical direction in the following situations. V0: If the flame is extinguished without dripping within 10 s V1: If the flame is extinguished without dripping within 30 s V2: If the flame is extinguished by dripping in 10 s. To determine fire properties, the UL flame rating complies with the following standards: IEC 60707, IEC 60695-11-10, IEC 60695-11-20, ISO 9772, and ISO 9773.

### Physical properties method and standards

Physical properties of manufactured resins using Heat Deflection Temp tests were investigated (HDT) ISO 75-A, ASTM 648, Hardness ASTM D2583, Volumetric shrinkage ASTM 3521, and Water absorption ASTM D570.

### Synthesis tetraallyloxysilane (TAS)

**Figure 1:**
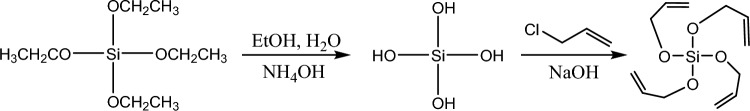
Synthesis of tetraallyloxysilane (TAS).

TAS was synthesized using the hydrolysis TEOS; in a 250 mL beaker equipped with a magnetic stirrer, TEOS/EtOH/H_2_O = 0.2/0.8/4 mol (41.51/36.73/71.75 g) was introduced at 25 °C. After adding drop by drop of 1 mL of an ammonia solution (25% by weight), the beaker was sealed with a plastic foil to prevent evaporation, and the solution was stirred for 12 h at 25 °C. The progress of the reaction was tested using TLC paper in an ethanol solvent until the completion of the TEOS reaction. The product was isolated by filtration and dried under a vacuum in a laboratory oven for 12 h at room temperature. The synthesized Si(OH)_4_ (40 g) was dissolved in THF (40 g) as the solvent and transferred into a two-neck round bottom flask equipped with an electric stirrer and a condenser under a nitrogen atmosphere. A sodium hydroxide solution (30% by weight, 1 mL) was gradually introduced into the reaction mixture, ensuring the temperature remained below 36 °C. The mixture was then cooled down to 0 °C using an ice bath. Afterwards, allyl chloride (1.70 mol, 130.66 g) was slowly added, drop by drop, into the flask while stirring for 3 h, keeping the temperature lower than 5 °C. Subsequently, the flask was heated at 55 °C and kept at this temperature for 12 h, after which the flask was cooled to room temperature. The solvent was removed under vacuum at room temperature, obtaining a clear, slightly viscous solution (Fig. 1).

FTIR (ν_max_, cm^−1^): Part D 3062 (–CH), 2872 (–CH_2_), 1604–1496 (–C=C) alkene bond, 1081 (–Si–CH_2_) 1081 (–Si–O–C_2_H_5_) (Fig. 5).

^1^H-NMR (CDCl_3_) δ (ppm): 4.37 (d, 8H, 4 × CH_2_), 5.17 (dd, 8H, 4 × =CH_2_ alkene), 5.80 (m, 4H, 4 × =CH alkene) (Fig. 7).

### Synthesis of unsaturated polyester resin (UPE)

**Figure 2:**
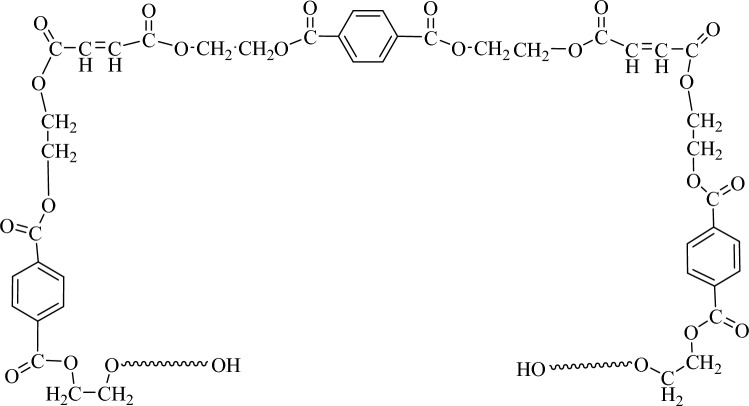
Synthesis of unsaturated polyester resin (UPE).

UPE was synthesized through the reaction between terephthalic acid (TPA), ethylene glycol (EG), and maleic anhydride (MA). A 500 mL round bottom flask with five necks, mechanical stirrer, thermal heater, thermometer, condenser, dropping funnel, and nitrogen gas injection tube were prepared. Nitrogen was fluxed in the flask for 30 min, and then TPA, EG, DBTO, and MA were introduced into the reactor and stirred for 1 h at 150 °C. The reactor was then heated at 185 °C at a 1 °C/min rate, continuing the nitrogen flux and the reaction for 3 h. Finally, a sample was collected with a spatula and tested through FT-IR and ^1^H-NMR. The reactor was cooled down at 90 °C, and, under stirring, a styrene solution was added to the reactor from the dropping funnel. The reaction continued for 1 h, then the product was collected. Table 1 reports the molar ratios of monomers, and Fig. 2 shows the schematic representation of the prepared resin before adding styrene.

**Table 1:** Synthesis of unsaturated polyester resin.

Mole ratio			
Sample glass pilot scale reproduction name	UPE	UPE I	UPE II
Compound			
EG	2.30	2.30	2.30
TPA	1.14	1.25	1.35
MA	0.76	0.76	0.76
DBTO	0.0012	0.0012	0.0012
Styrene	1.74	1.81	1.89

FTIR (ν_max_, cm^−1^): Part D 3541 (–OH), 2963 (–CH_3_), 2876 (–CH_2_), 1606–1471 (–C=C) alkene and aromatic bond, 1239 (–C–O–C), 1133 (–C–O) (Fig. 6).

^1^H-NMR (CDCl_3_) δ (ppm): 3.72 (t, 6H, 3 × CH_2_), 4.29 (t, 4H, 2 × CH_2_), 4.62 (t, 8H, 4 × CH_2_), 4.89 (t, 8H, 4 × CH_2_), 5.64 (s, 2H, 2 × OH), 6.88 (d, 4H, 4 × =CH alkene), 7.28 (s, 12H, 6 × =CH aromatic) (Fig. 8).

#### Preparation of allyloxysilane-unsaturated polyester (AUPE)

**Figure 3:**
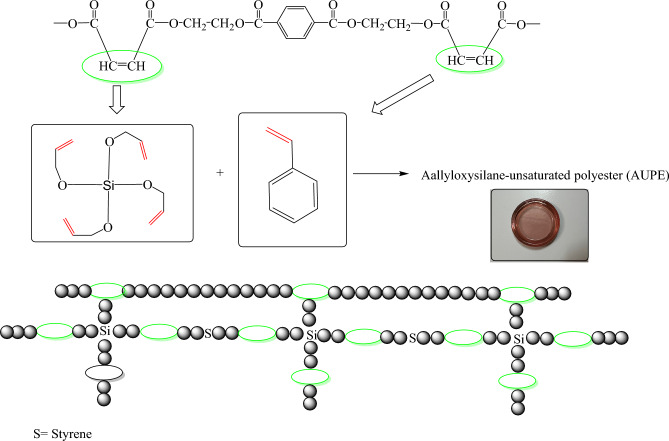
Preparation of allyloxysilane-unsaturated polyester (AUPE).

The allyloxysilane-unsaturated polyester resin was prepared by reaction of UPE with different amounts of tetra allyloxysilane (TAS) according to the data reported in Table 2 and Fig. 3. The UPE and TAS were inserted in a beaker and stirred for 5 min at room temperature; then, the product was evaluated in the following way.

**Table 2:** Preparation of allyloxysilane-unsaturated polyester.

Sample	UPE (%)	Tetraallyloxysilane (TAS) (%)	Benzoyl peroxide (%)	Cobalt octoate (%)
AUPE	97.5	0	2	0.5
AUPE I	77.5	20	2	0.5
AUPE II	67.5	30	2	0.5

#### Preparation of allyloxysilane-unsaturated polyester (AUPE) Film

**Figure 4:**
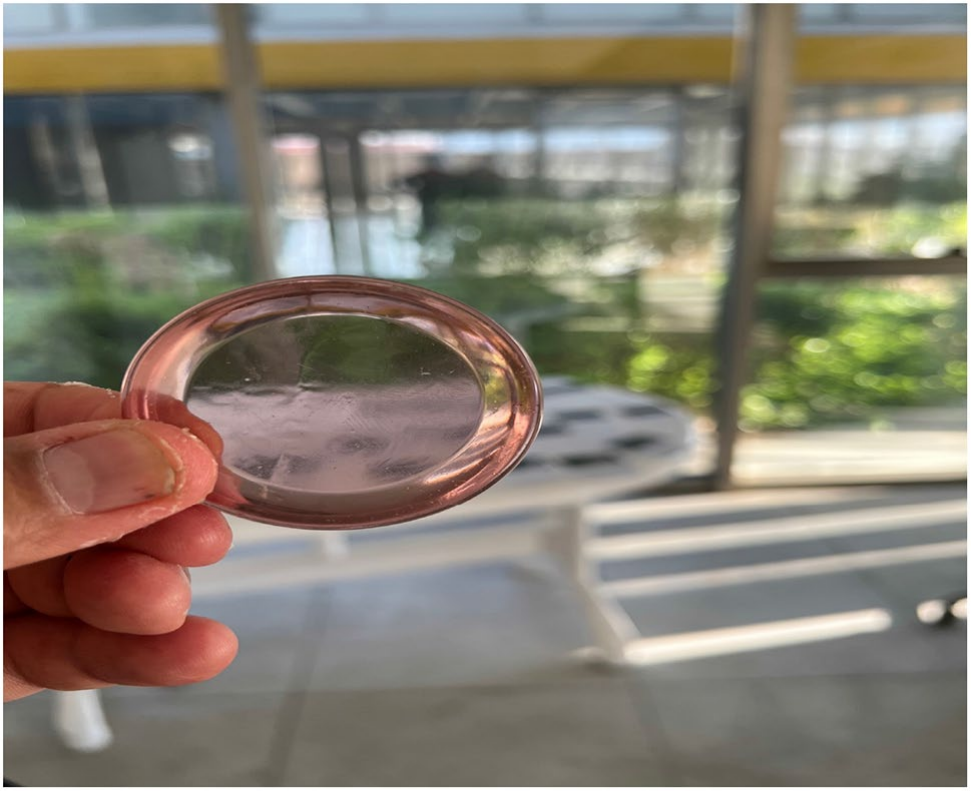
Allyloxysilane-unsaturated polyester (AUPE) film.

The produced films are first mixed with 20 g of resin mixture with 10% benzoyl peroxide and 10% cobalt octoate with the proportions mentioned in Table 2 and stirred for 1 min at room temperature with a glass spatula and then added to the template container. A metal can lid (approximately 5 cm in diameter) was used as a mold and dried at laboratory temperature for 12 h. The thickness of the prepared film is 4 mm. An example of the prepared film is shown in Fig. 4.

## Results and discussion

Tetraallyloxysilane was synthesized in two steps: the first step was the hydrolysis of TEOS to form Si(OH)_4_, and in the second step, the Si(OH)_4_ through SN^2^ reaction with allyl chloride in the presence of NaOH gave the expected TAS. Also, the polyester resin was synthesized through the reaction between TPA and bi-functional alcohol (EG) in 5 h, obtaining an utterly transparent resin. Then, a mixture of tetraallyloxysilane and polyester resin reacted to obtain a transparent, helpful film for various applications, such as wood surface coatings. The intermediate and the final compounds were characterized using different analyzes.

### FTIR analysis

**Figure 5:**
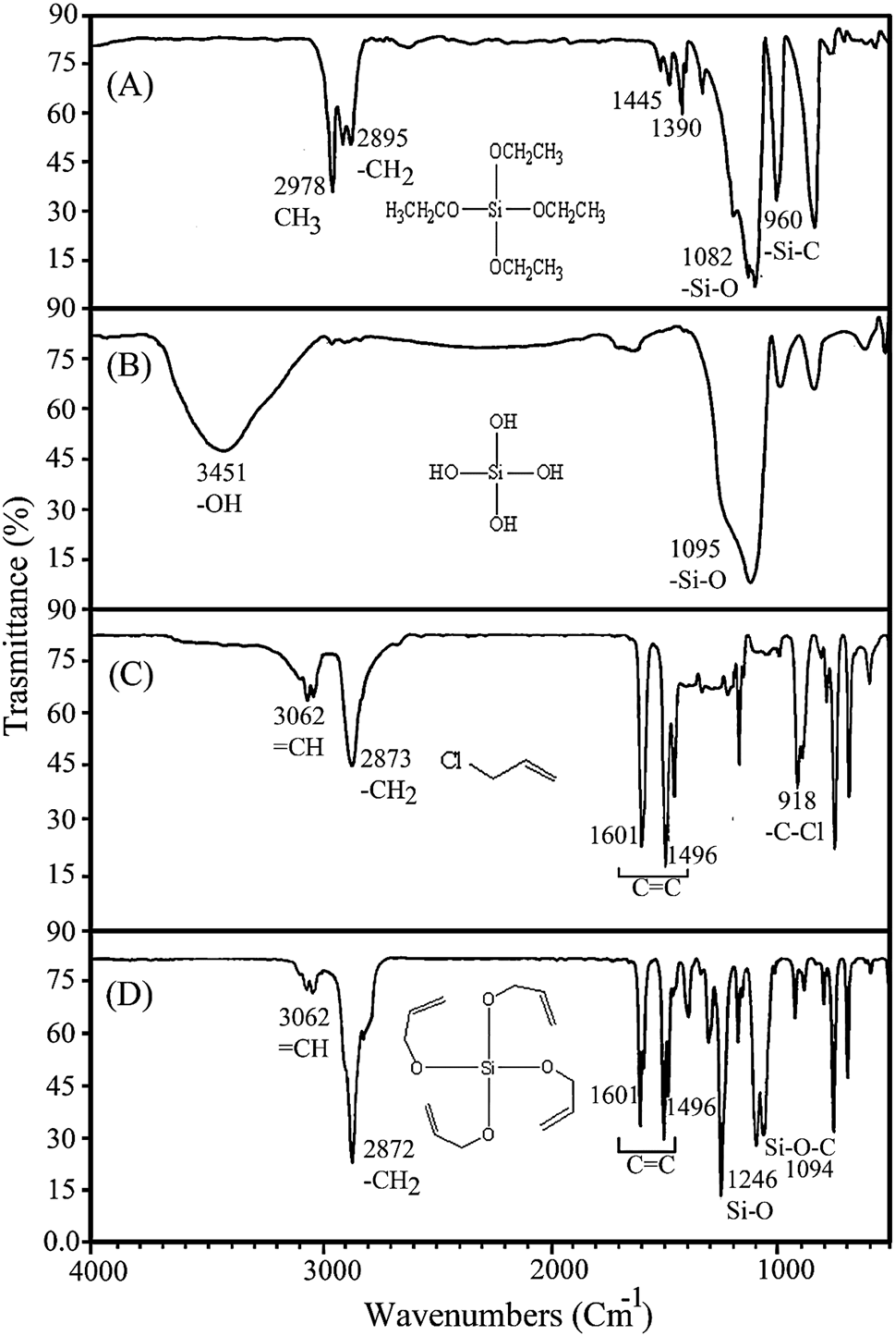
FTIR spectra of TEOS (**A**), hydrolysis spectrum of TEOS (**B**), allyl chloride (**C**), and tetraallyloxysilane (**D**).

The FTIR spectrum of Fig. 5 shows the successful synthesis of Tetraallyloxysilane in its four intermediates A, B, C, and D: (A) shows a spectrum related to TEOS; (B) shows the absence of CH_2_ and CH_3_ peaks at 2978 and 2895 cm^−1^ and the presence of a broad peak attributed to –OH groups at 3451 cm^−1^ and peak Si–O in the region of 1095 cm^−1^ confirming the successful hydrolysis of TEOS. The spectrum (C) shows pure allyl chloride, while the spectrum (D) shows the synthesized tetraallyloxysilane where asymmetric and symmetric C–H stretching vibrations of methylene groups were present at 3062 and 2872 cm^−1^ and C=C stretching vibrations at 1601 and 1496 cm^−1^ while the peak at 1246 and 1094 cm^−1^ are attributed to the stretching vibrations of Si–O and Si–O–CH groups. Also, the C–Cl peak at 918 cm^−1^ indicates the successful synthesis of tetraallyloxysilane.

**Figure 6:**
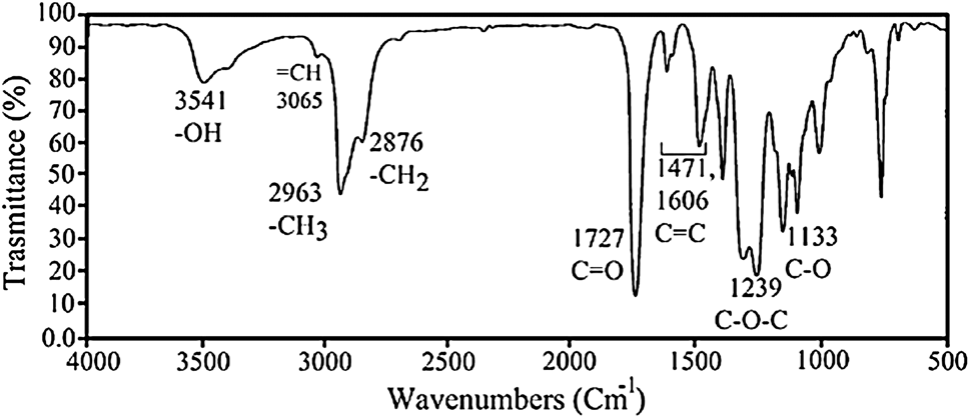
FTIR spectrum of synthesis unsaturated polyester resin (UPE).

Figure 6 shows the FTIR spectrum related to the synthesis of unsaturated polyester resin (UPE). As shown in Fig. 2, the formation of the product is confirmed by the presence of the stretching –OH groups in the 3541 cm^−1^ regions with a broad peak. The peak related to the =CH groups is present at 3065 cm^−1^, attributed to hydrogen attached to the double bonds, while the absorption of aromatic C=C bonds is present at 1471 and 1606 cm^−1^. Methylene and methyl groups show –CH stretching absorption at 2963 and 2876 cm^−1^ regions. The carbonyl ester group C=O shows a sharp and broad peak in the region of 1727 cm^−1^. C–O–C and C–O showed one peak with vigorous intensity at 1239 cm^−1^ and one with moderate intensity at 1133 cm^−1^, showing the ester compound’s successful synthesis.

### ^1^H-NMR analysis

One of the most efficient techniques for analyzing the structure of organic compounds and polymers is ^1^H-NMR spectroscopy.

**Figure 7:**
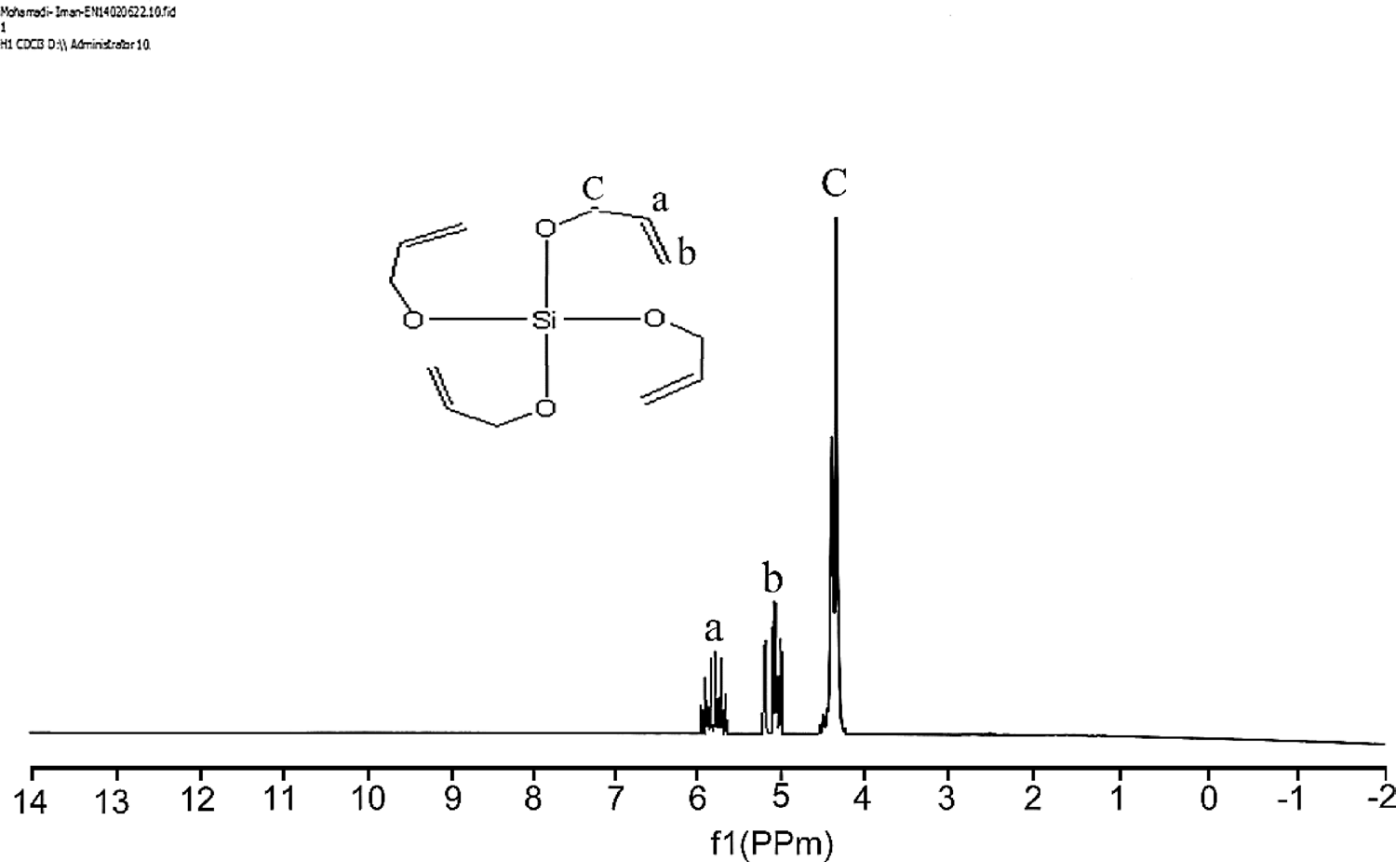
^1^H-NMR spectrum of tetraallyloxysilane.

**Figure 8:**
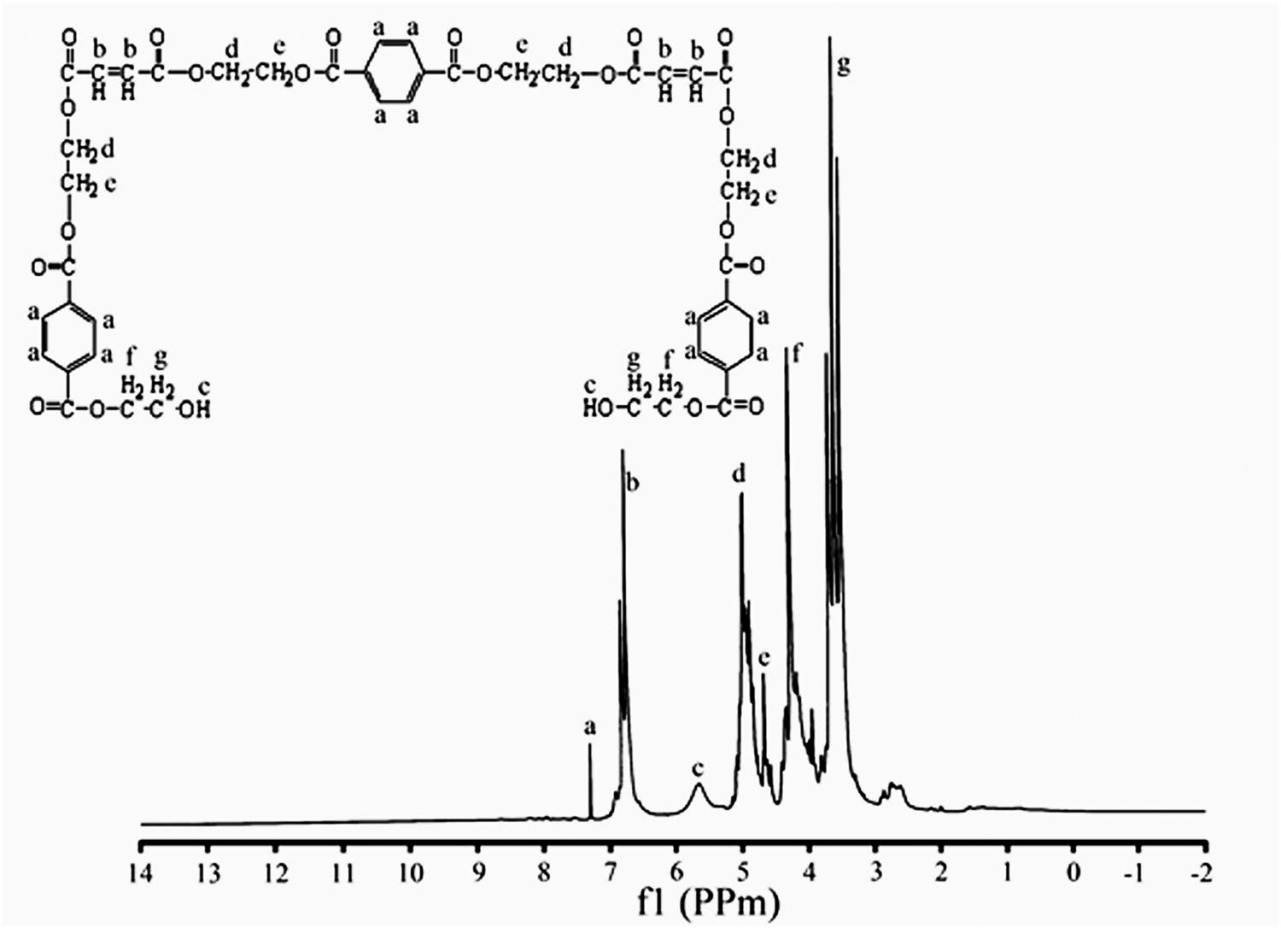
^1^H-NMR spectrum of unsaturated polyester resin (UPE).

The ^1^H-NMR spectrum of tetraallyloxysilane (Fig. 7) showed resonances at δ (ppm): 4.37 (d, 8H, 4 × CH_2_), 5.17 (dd, 8H, 4 × =CH_2_ alkene), 5.80 (m, 4H, 4 × =CH alkene). The ^1^H-NMR spectrum of the synthesized unsaturated polyester resin (UPE) (Fig. 8) showed resonances at δ (ppm): 3.71 (t, 4H, HO–CH_2_^*^–CH_2_), 4.35 (t, 4H, HO–CH_2_–CH_2_^*^), 4.74 (t, 8H, –O–CH_2_^*^–CH_2_–O), 4.91 (t, 8H, –O–CH_2_–CH_2_^*^–O), 5.65 (s, 2H, ^*^HO–CH_2_), 6.88 (d, 4H, CH_2_^*^ =CH_2_^*^), 7.29 (s, 4H, =CH^*^ aromatic protons) (Fig. 8).

### The molecular weight of UPE

**Figure 9:**
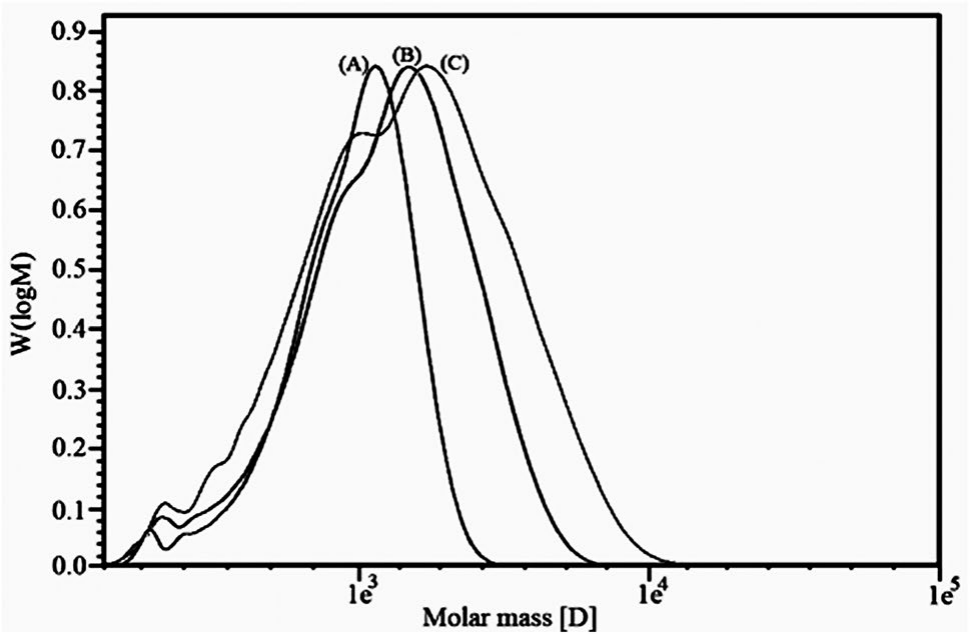
The molecular weight curves of UPE (**A**), UPE I (**B**), and UPE II (**C**).

Its molecular weight is a critical parameter affecting the performance and applications of unsaturated polyester resins. Unsaturated polyester resins can be used as surface coatings, laminates, and composites. The molecular weight resulting from the polymerization process affects the viscosity of the resin, the curing characteristics, and the mechanical properties. Chemical resistance and mechanical strength for harsh applications such as wood coatings and parts of marine vessels and automotive parts are improved by molecular weight. Precise molecular weight control is necessary to balance processability and final performance in various applications. In this study, different molar ratios among TPA, EG, and styrene (Table 1) were employed to test their influence on the molecular weight and density of the polymer. Table 3 reports the Mw and density of the obtained unsaturated polyester resins, while Fig. 9 shows the curves obtained from the GPC analysis of the same polymers. These data indicate that increasing the molar mass of terephthalic acid increases the molecular weight and the density of the synthesized UPE.

**Table 3:** Mw and density of different UPE.

Sample name	UPE	UPE I	UPE II
Tests			
Molecular weight (Mw) g/mol	1625	1876	2531
Density (g/cm^3^)	1.080	1.109	1.129

### Thermal properties

**Figure 10:**
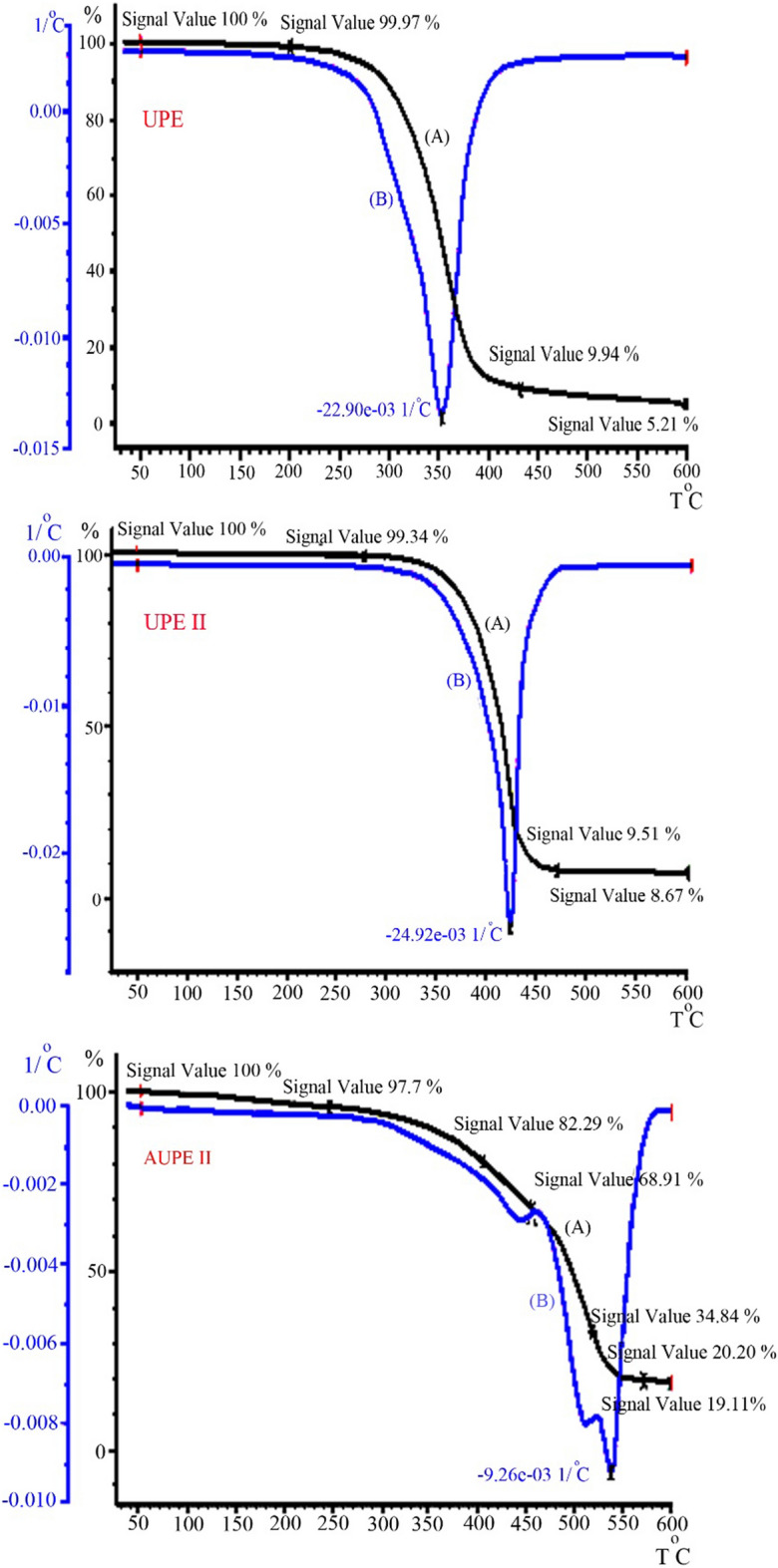
TGA/DTG of UPE, UPE II, AUPE II.

TGA/DTG/DSC analyzes were used to study the thermal properties of the prepared UPE. Under a flux of 60 mL/min of inert gas (nitrogen), thermal analysis curves were collected, detecting the weight loss of the sample (W) as a function of temperature (T) with a temperature increase rate of 10 °C/min from room temperature to 600 °C. Three samples were thermally analyzed: UPE resin, UPE II resin without tetraallylsilane, and AUPE resin where UPE was previously reacted with tetraallyloxysilane (Fig. 10).

The first weight loss in all samples was observed in the 200–300 °C range and was attributed to the weight loss of the absorbed water. Figure 10, related to the UPE resin, showed at 350 °C a second weight loss attributed to the decomposition of the ester compound. At the end, 5.21% of the starting sample remained at 600 °C. In UPE II resin (Fig. 10), a second weight loss occurs at 412 °C. This weight loss was also attributed to the breakdown of the ester compound. The higher temperature required for this pyrolysis is attributed to the higher percentage of terephthalic acid in the polymer. The residue after the heating at 600 °C was 8.67%. Figure 10 reports the TGA/DTG related to AUPE II, the resin containing tetraallyloxysilane and styrene, showed better temperature resistance, and its diagram contains three weight loss. The first peak is attributed to the destruction of the polyester structure, which starts at 405 °C; the second peak starts at 456 °C, which is related to the loss of styrene moiety, and the third peak started at 519 °C, was associated with the loss of tetraallyloxysilane moiety and at the residual product was 19.11%.

**Figure 11:**
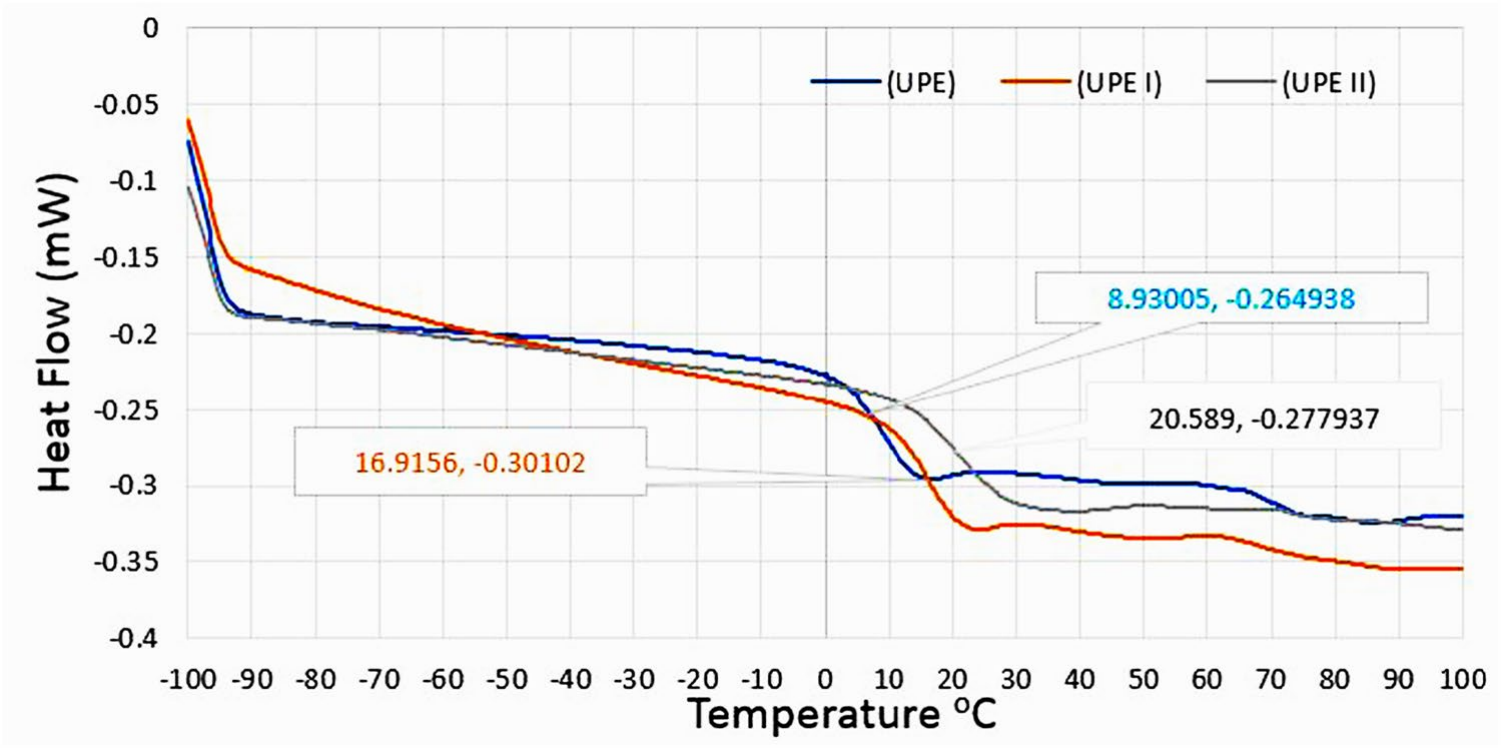
DSC curves of UPE, UPE I, and UPE II resins.

In the DTG diagram, the rate of weight change is plotted as a function of temperature. The decrease in weight of the sample is shown on the abscissa axis in a downward direction, and the increase in weight is shown in an upward direction. The UPE sample showed the average temperature of degradation and the most significant weight loss as a sharp peak in the region of 355 °C. The corresponding temperature for the sample UPE II was 445 °C, and the sample AUPE was 538 °C. The DSC analysis showed the temperature difference (ΔT) between the inert reference and the sample as a temperature (T) function while the materials are under a thermal control program. Figure 11 shows the DSC curves of UPE, UPE I, and UPE II resins, where the curves go below the baseline due to an endothermic process, and also shows the average glass transition point in UPE at 8.93 °C, UPE I at 16.91 °C and UPE II at 20.58 °C. As can be seen, with the increase in the molecular weight of the polymer and the presence of a higher amount of terephthalic acid, the glass transition point increases, and the temperature resistance of the polymer increases.

### Flammability test on AUPE

**Figure 12:**
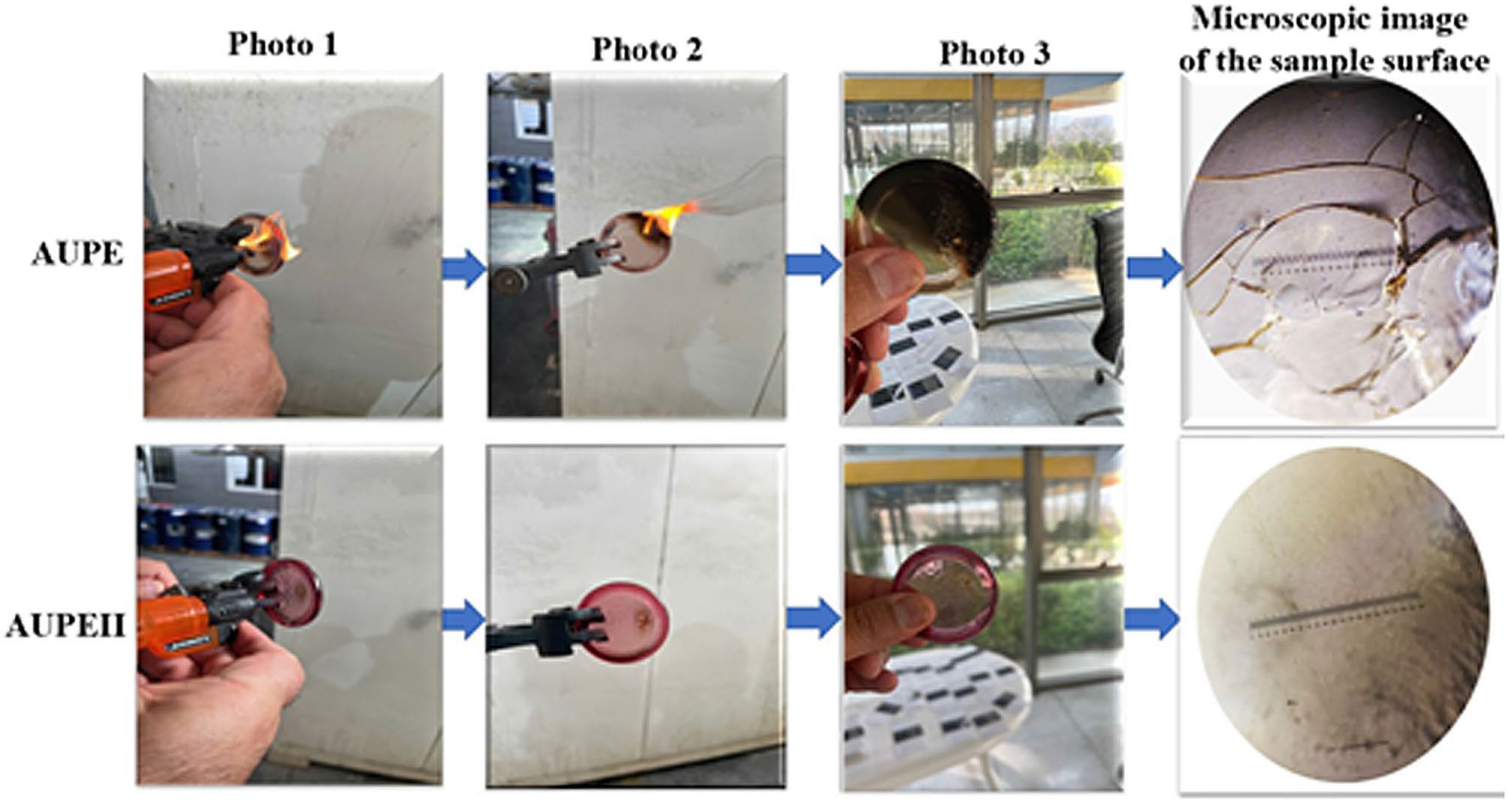
Flammability tests of AUPE and AUPE II.

**Figure 13:**
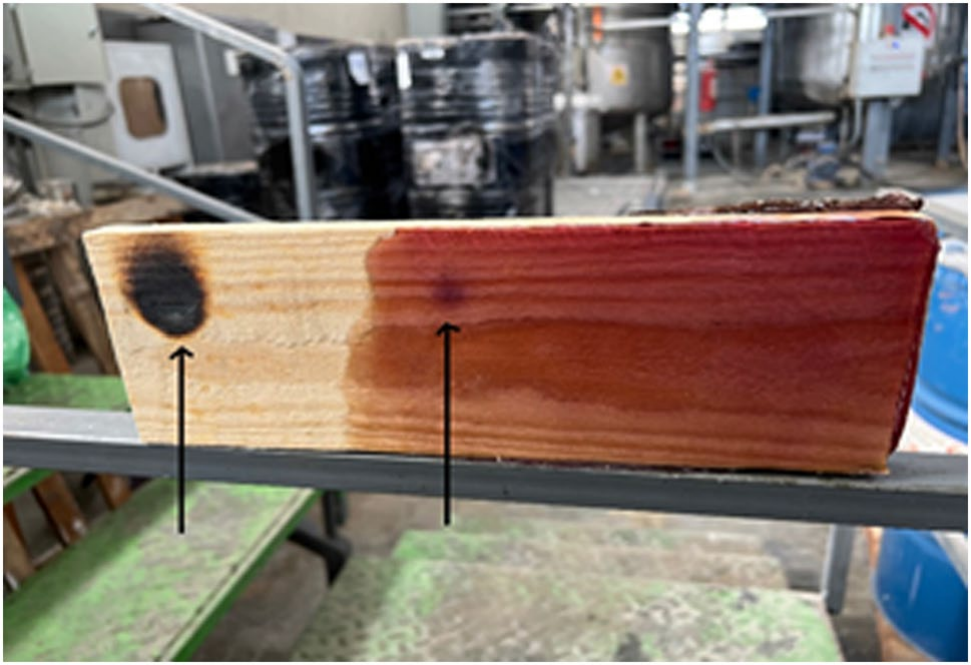
Flammability of wood coated and not-coated with AUPE resin.

**Figure 14:**
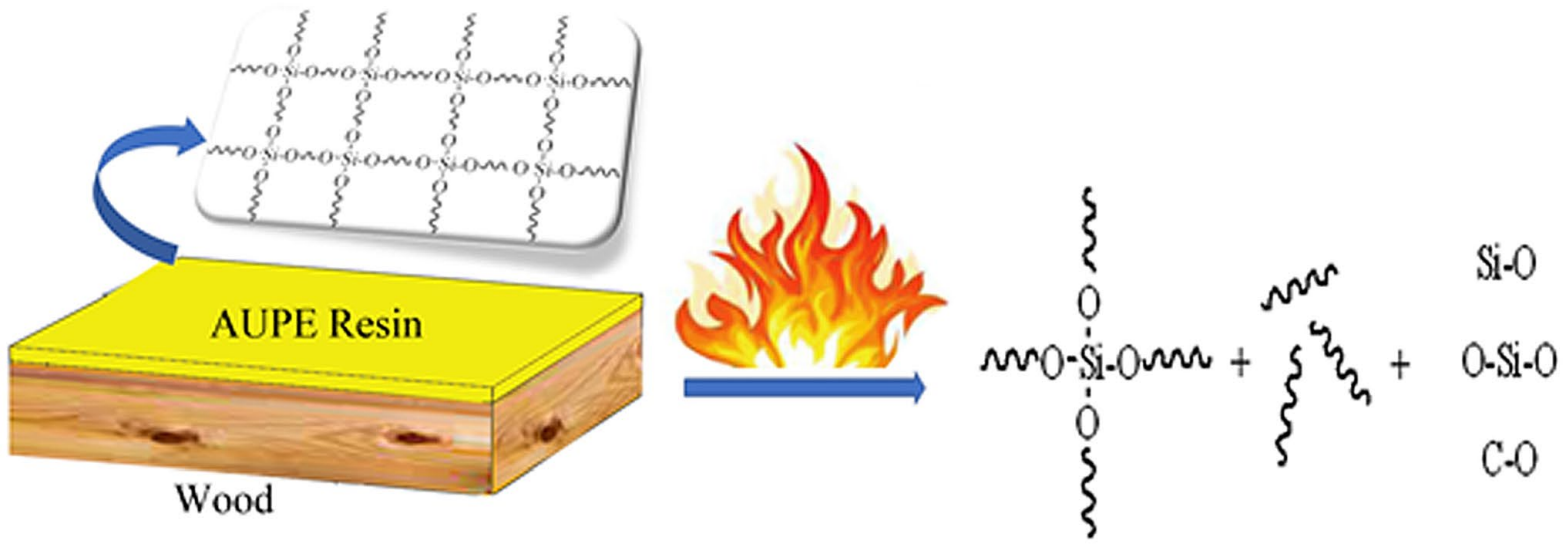
Flam destruction mechanism of wood coated with AUPE resin.

The test samples were placed in the presence of an open flame with a temperature of 1200 °C from a distance of 2 cm for 10 s, and their thermal behavior was checked (Fig. 12); photo 2 shows that the AUPE did not comply with the UL94 standard and was fully ignited and flame did not stop after 30 s. However, photo 2 shows that AUPE II fully complies with the UL94 standard, and even the flame did not ignite continuously and was extinguished in less than 10 s. This different behavior may be attributed to the presence of TAS in the AUPE II sample. Also, the microscopic images taken with a 10-micron 0.01 mm sheen microscope Fig. 12 show that the UPE form is wholly cracked and destroyed. However, the AUPE II image showed that the plastic remained fully intact. Also, according to the BS 476 standard of India and the United Kingdom, a coating of AUPE II resin was applied in three layers 1 mm thin (all together or each one) on a piece of wood and placed in the presence of a 1200 °C flame for 3 s at a distance of 2 cm, as shown in Fig. 13. A surface of 3 cm^2^ was ignited and damaged on the untreated sample, while in the one coated with AUPE II, the surface's color was slightly changed, but no destruction was seen. If we continue with this standard for a longer time, the working mechanism of the applied resin coating will be shown in Fig. 14; as you can see, the main base of the polymer remains at a temperature of more than 700 °C^46^.

### Physical properties

**Figure 15:**
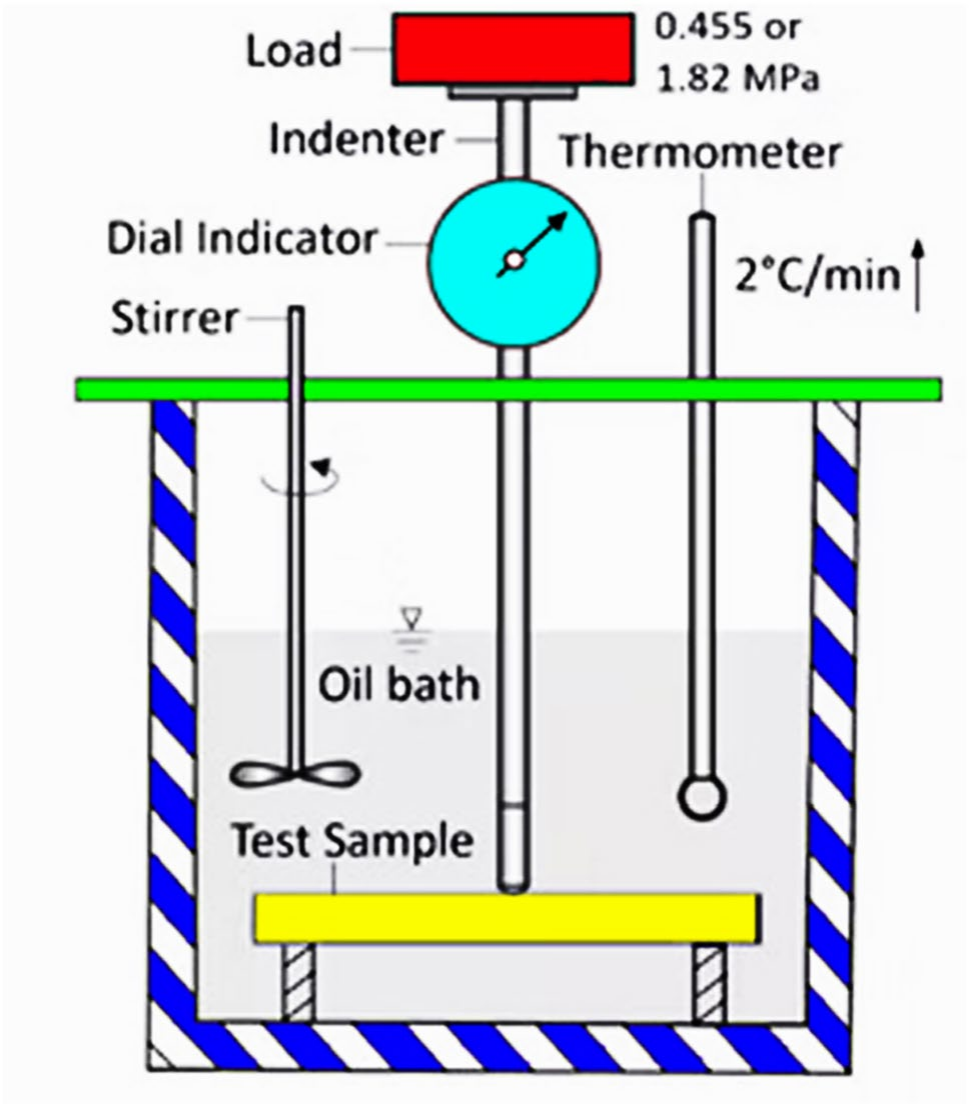
Schematic of the HDT measuring device.

The polymer's resistance to deformation under a particular load at high temperature is called thermal distortion or thermal deflection temperature (HDT), the temperature at which a polymer rod is flexed by 0.25 mm. Practically, the hardness of the polymer is measured by increasing the temperature up to a flexion. In this method, according to Fig. 15, first, a polymer rod is made by a unique machine mold from three resins: AUPE, AUPE I, and AUPE II, with a length of 127 mm, width and depth of 13 mm, and immersed in oil one by one, with increasing temperature at about 2 °C/min, the force applied and adjusted to 1.82 MPa created a pressure on the middle point of the strip. The temperature increased progressively until the rod was deformed by 0.25 mm and was recorded as the HDT temperature. Table 4 shows that with the increase of tetraallyloxysilane in the polymer, the HDT of the resin increased from 77 °C for AUPE 77 °C, to 105 °C for AUPE II.

**Table 4:** Physical properties of AUPE, AUPE I, and AUPE II.

Physical properties	STM	Unit	AUPE	AUPE I	AUPE II
Heat Deflection Temp. (HDT)	ISO 75-A, ASTM 648 ≥ 76	°C	77	89	105
Hardness	D2583 ≥ 38	Barcol 934-1	38	47	55
Volumetric shrinkage (%)	ASTM 3521 ≤ 9%	–	7.011	4.347	2.222
Water absorption (%)	ASTM D570 ≤ 0.4%	mg	0.43	0.28	0.12

**Figure 16:**
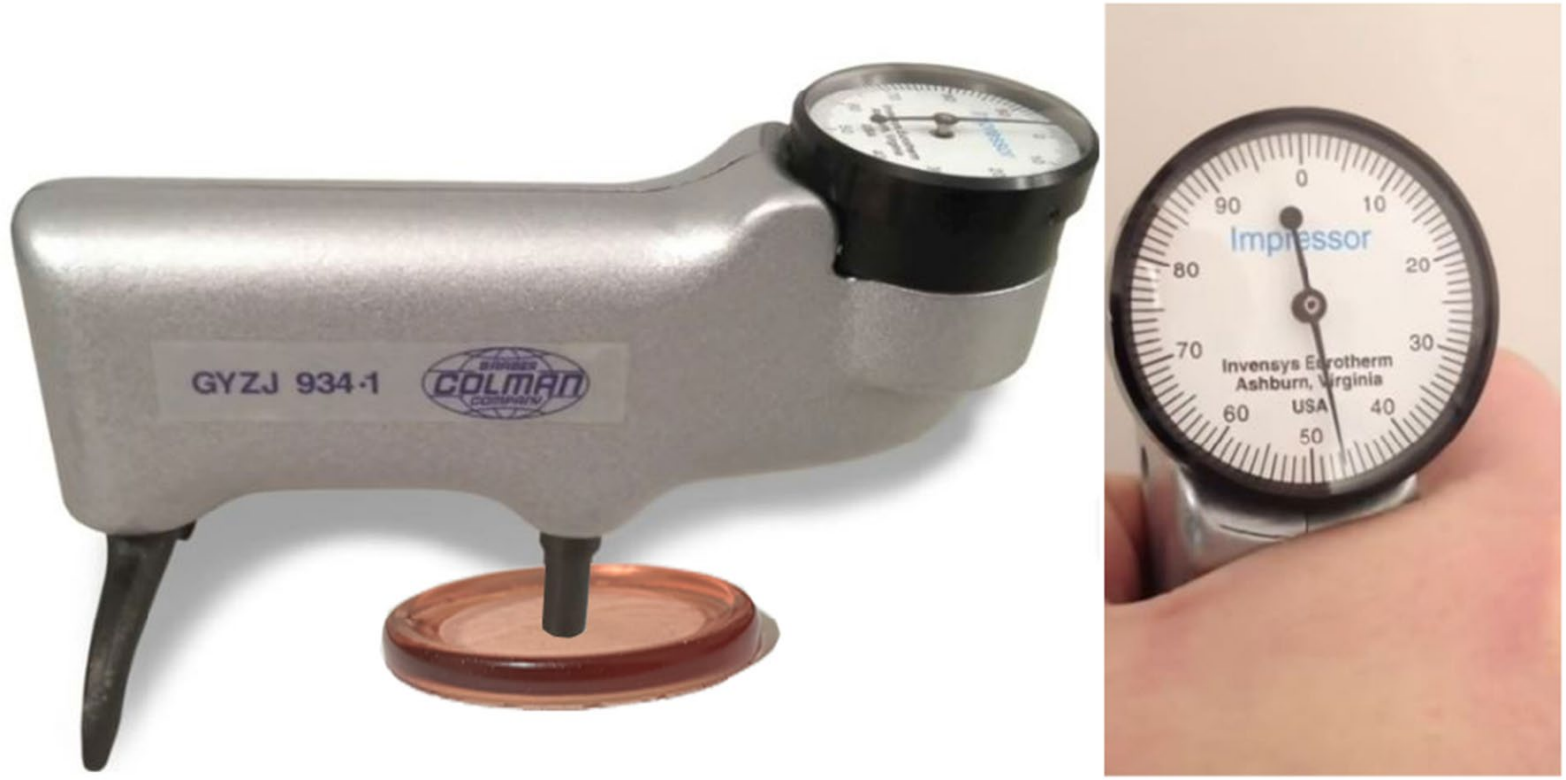
Barkel hardness tester methods.

The Barkle method is one of the methods employed to evaluate the hardness of materials, especially plastic materials, composites, and resins. This hardness test is determined by the indentation depth in the material by a sharp steel point under the spring loading force on the polymer. In this method, a sample tablet was realized for AUPE, AUPE I, and AUPE II polymers, and the sample was placed under the teeth of the Barkel hardness tester, as shown in Fig. 16. A uniform vertical pressure was applied to the sample with the right hand until the pointer pointed to reach the maximum, and the indicated number was recorded. The depth of penetration is converted into absolute Barkel numbers. Table 4 shows Barkel numbers obtained for AUPE, AUPE I, and AUPE II samples 38, 47, and 55, respectively, which indicates the higher hardness of the samples AUPE II containing a higher amount of tetraallyloxysilane and the lower value in the AUPE sample without tetraallyloxysilane.

### Volumetric shrinkage

Polymerization shrinkage is calculated using molding, baking, and measuring processes according to ASTM 3521 standards. According to the data reported in Table 2, the sample was prepared using a metal mold with a diameter of 45 mm and a depth of 4 mm. A polymer solution was inserted in the mold. After drying, the length of the mold is measured (the metal mold does not change its measure), and the length of the dried sample is subtracted from the length of the mold; the length of contraction is obtained, and the length of contraction is expressed as a percentage of the length of the metal mold.

$$\% {\text{ mold length }} = \, \left( {{\text{mold length }} - {\text{ sample length}}} \right) \, \times {1}00/{\text{mold length}}$$

**Figure 17:**
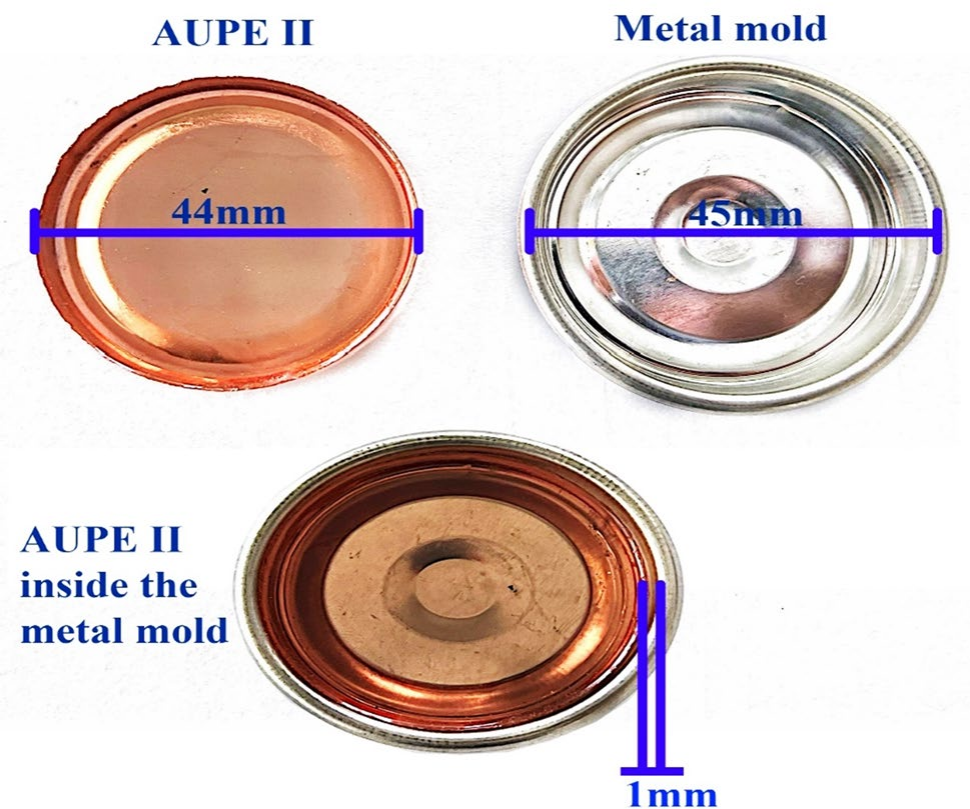
Volumetric shrinkage of AUPE II Resin.

Figure 17 shows the shrinkage percentage of AUPE II resin; as can be seen, the length of the metal mold is 45 mm, and the sample length is 44 mm. Therefore, the difference between these two tests shows the size of contraction, which is 2.222%. Thus, Table 4 shows that the shrinkage percentage decreased with the increase of tetraallyloxysilane.

### Water absorption

The ideal penetration of water into the polymer is called water absorption. The relative rate of water absorption of plastics was measured according to the ASTM D570 standard. In this method, a polymer tablet with a thickness of 3.2 mm and a diameter of 45 mm was made, and then the sample was placed in an oven with a constant temperature of 105–110 °C to obtain a dried sample. After cooling the sample in a desiccator, it was weighed, and the value was recorded as the dry weight of the sample, which was approximately 1 mg. After weighing about 1 mg, the dried sample was placed in a container containing distilled water heated at 23 ± 1 ºC and left for 24 h. At the end, the sample was removed from the distilled water, and all the sample surfaces were dried with a cloth and weighed. The resulting weight was recorded as wet weight.

Finally, the water absorption percentage of the samples was calculated using the following formula.

$$Water\;absorption\;percentage = \frac{Wetweight - dryweight}{{dryweight}} * 100$$

**Figure 18:**
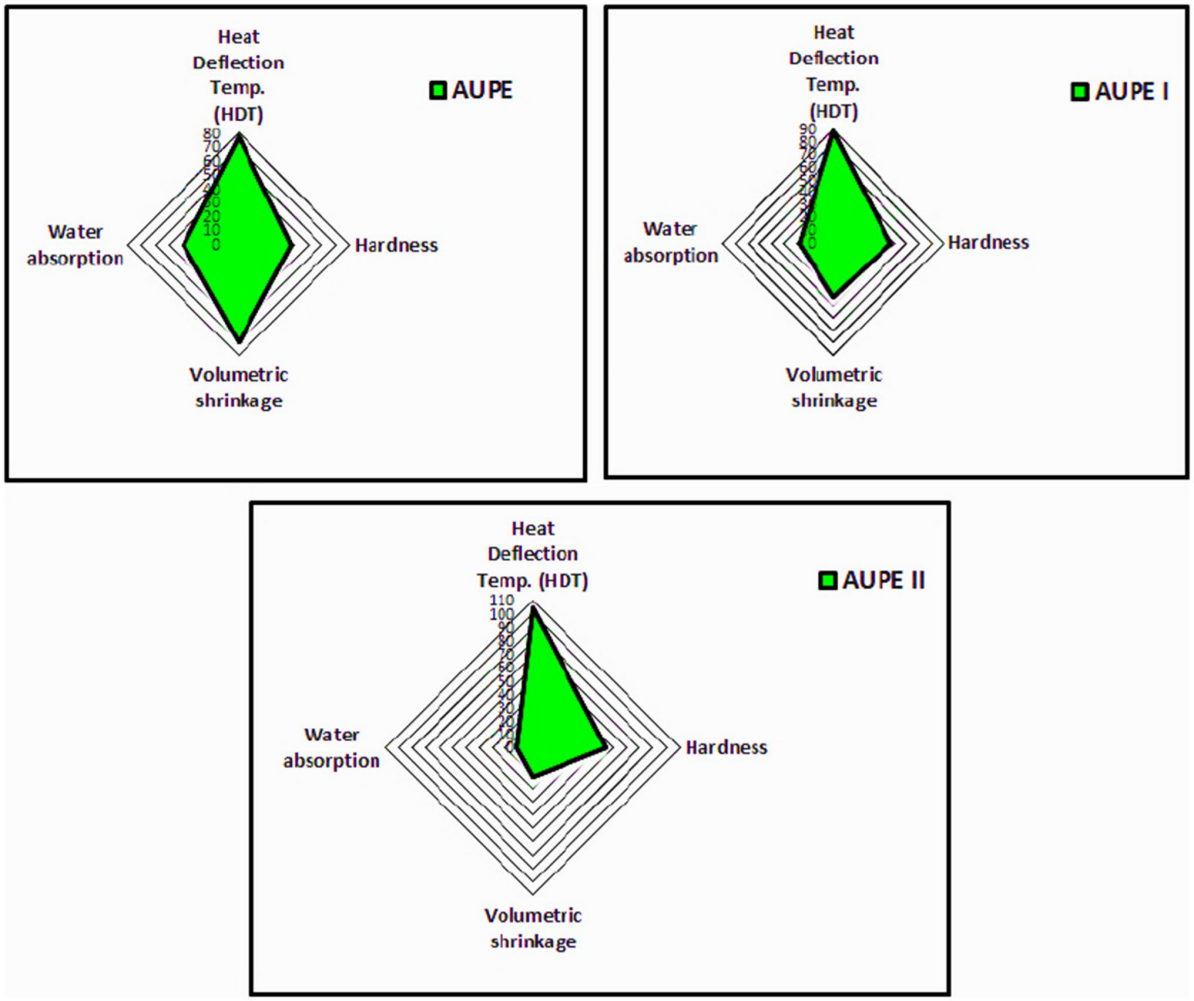
Performance evaluation diagram of physical properties for films made by AUPE, AUPE I, and AUPE II.

As you can see in Table 4, the water absorption of AUPE I and AUPE II polymer samples containing tetraallyloxysilane is 0.28 and 0.12%, respectively, lower than the water absorption of AUPE and has shown better performance. Table 2 and Fig. 18 also show that AUPE II performs best with the lowest amount of water absorption, the lowest volumetric shrinkage, and the highest hardness. The water absorption behavior of polyester resin, such as AUPE insulation, is determined by measuring the change in weight at different relative humidity. As you know, silicone compounds are used as hydrophobic compounds due to their low solubility in water and good dispersing properties, so with the increase in the percentage of TAS in AUPE II, Fig. 18 shows the lowest amount of water absorption^47,48^. Curing using TAS has caused the tetrahedral network of branched polyester so that silicon is placed in the center of this curing, so the resistance to volumetric shrinkage, hardness, and HDT increases.

## Conclusions

Wood is increasingly being used in construction worldwide for interior and exterior decoration. However, its flammability requires treatment with specific materials. Heat-resistant polyester resins are crucial for wood coatings, providing durability and protection against environmental stress. This project aims to synthesize a polyester resin using terephthalic acid for durability and enhancing thermal resistance with tetraallyloxysilane. The synthesis of unsaturated polyester resin using TPA was successful according to FT-IR and ^1^H-NMR data. The density and molecular weight of the polymer increased, controlled by the GPC test. Tetraallyloxysilane was synthesized through hydrolysis of TEOS and confirmed by FT-IR and ^1^H-NMR data. The final AUPE resin was obtained by reacting UPE with TAS. Increasing the ratio of tetraallyloxysilane to polyester resin improved the thermal resistance. The glass transition temperature increased with molecular weight, reaching 20.58 °C in AUPE II. The AUPE samples showed increased residual weight and a higher maximum weight loss temperature. AUPE II exhibited superior physical properties, including water absorption, shrinkage, hardness, and HDT. It provides excellent protection against ignition when applied to wood surfaces, making AUPE resins ideal for ensuring longevity, durability, and fire resistance in buildings.

## Data Availability

All data have been given in the article.
